# Improved Volitional Recall of Motor-Imagery-Related Brain Activation Patterns Using Real-Time Functional MRI-Based Neurofeedback

**DOI:** 10.3389/fnhum.2018.00158

**Published:** 2018-04-24

**Authors:** Epifanio Bagarinao, Akihiro Yoshida, Mika Ueno, Kazunori Terabe, Shohei Kato, Haruo Isoda, Toshiharu Nakai

**Affiliations:** ^1^Brain & Mind Research Center, Nagoya University, Nagoya, Japan; ^2^Department of Radiological Sciences, Nagoya University Graduate School of Medicine, Nagoya University, Nagoya, Japan; ^3^NeuroImaging and Informatics Lab, National Center for Geriatrics and Gerontology, Obu, Japan; ^4^Graduate School of Engineering, Nagoya Institute of Technology, Nagoya, Japan

**Keywords:** real-time fMRI, motor imagery, neurofeedback, brain machine interface, functional MRI, support vector machine

## Abstract

Motor imagery (MI), a covert cognitive process where an action is mentally simulated but not actually performed, could be used as an effective neurorehabilitation tool for motor function improvement or recovery. Recent approaches employing brain–computer/brain–machine interfaces to provide online feedback of the MI during rehabilitation training have promising rehabilitation outcomes. In this study, we examined whether participants could volitionally recall MI-related brain activation patterns when guided using neurofeedback (NF) during training. The participants’ performance was compared to that without NF. We hypothesized that participants would be able to consistently generate the relevant activation pattern associated with the MI task during training with NF compared to that without NF. To assess activation consistency, we used the performance of classifiers trained to discriminate MI-related brain activation patterns. Our results showed significantly higher predictive values of MI-related activation patterns during training with NF. Additionally, this improvement in the classification performance tends to be associated with the activation of middle temporal gyrus/inferior occipital gyrus, a region associated with visual motion processing, suggesting the importance of performance monitoring during MI task training. Taken together, these findings suggest that the efficacy of MI training, in terms of generating consistent brain activation patterns relevant to the task, can be enhanced by using NF as a mechanism to enable participants to volitionally recall task-related brain activation patterns.

## Introduction

A growing number of neuroimaging studies have shown that brain regions activated during an overt motor task are also activated during motor imagery (MI), a covert cognitive process where an individual mentally simulates an action but without any body movement (Grèzes and Decety, 2000; Hétu et al., 2013). Key anatomical structures typically activated in both overt movement and MI include the primary motor cortex (M1), supplementary motor area (SMA), premotor cortex (PMC), prefrontal cortex (PFC), posterior parietal cortex (PPC), cerebellum and basal ganglia (O’Shea and Moran, 2017; Tong et al., 2017). Aside from activating similar cortical areas, overt movement and MI have also been shown to share cortical networks, although networks specific to each task were also reported (Sharma and Baron, 2013). These findings are aptly summed up by the mental simulation hypothesis (Jeannerod, 2001; O’Shea and Moran, 2017) which states that overt movement and MI are essentially based on the same processes. To account for the absence of actual movement in MI, Guillot et al. (2012) argued that MI also includes motor execution commands but are inhibited at some level of the motor system.

The notion that mental practice via MI could trigger activation in relevant motor areas raises the potential of MI as an effective neurorehabilitation tool to improve motor functions. As such, MI training has been employed in sports science to improve athletes’ performance (Feltz and Landers, 1983). The potential use of MI training as a no-cost, safe, and easy way to improve or preserve motor function in the elderly has also been examined (Saimpont et al., 2013). MI is also popular as a neurorehabilitation technique to enhance motor recovery following stroke (see relevant reviews in Jackson et al., 2001; Page, 2001; Sharma et al., 2006; de Vries and Mulder, 2007). Some studies have also shown that MI provides additional benefits to conventional physiotheraphy (Zimmermann-Schlatter et al., 2008) and can be used to identify potential sources of residual functional impairment in well-recovered stroke patients (Sharma et al., 2009a,b). However, recent findings have also shown that MI training does not enhance motor recovery in patients in early post stroke (Ietswaart et al., 2011). Thus, the efficacy of MI training for neurorehabilitation still remains unclear.

For MI to be effective, participants need to consistently generate activation patterns involving relevant brain regions during the training. This entails the ability to volitionally recall relevant MI-related brain activation patterns. An effective mechanism to evaluate MI task performance will also be needed (Sharma et al., 2006). To mitigate this problem, recent approaches employed brain-computer/brain-machine interfaces (BCI/BMI) in conjunction with MI to provide online contingent sensory feedback of the brain activity during rehabilitation training. In these approaches, BCI/BMI systems actively decode brain activity and display the outcome to the user to create feedback that is reflective of task performance. The addition of BCI in neurorehabilitation has been shown effective in improving clinical parameters of post-stroke motor recovery (Ramos-Murguialday et al., 2013; Ono et al., 2014; Frolov et al., 2017), suggesting the importance of providing feedback information during training to improve rehabilitation outcomes.

The importance of feedback in learning to volitionally control different aspects of brain activity has also been demonstrated in several neuroimaging studies. In particular, neurofeedback (NF) based on real-time functional magnetic resonance imaging (fMRI) has been shown effective in training participants to control brain activity within circumscribed brain regions (Posse et al., 2003; deCharms et al., 2005; Caria et al., 2007, 2010; Sulzer et al., 2013), control connectivity measures between regions (Koush et al., 2013, 2015), and induce multi-voxel activation patterns (Shibata et al., 2011). In this study, we systematically examined whether participants could volitionally recall MI-related brain activation patterns when guided using NF during training. The participants’ performance was compared to that without NF. Two recent studies on MI have shown that NF could significantly enhance task-specific brain activity compared to no NF (Zich et al., 2015; Perronnet et al., 2017). Based on these findings, we hypothesized that with NF during an extended MI task training, participants would be able to consistently generate the desired brain activation pattern associated with the MI task compared to that without NF. We assessed the consistency of recalled brain activation using the performance of classifiers trained to discriminate MI-related activation patterns. We further compared brain activations during MI with and without NF to investigate the neural mechanisms associated with NF during MI tasks training. For this, we developed a BMI system based on real-time fMRI that employed MI tasks to manipulate the arm movement of a small humanoid robot. The robot’s arm movement was then used to provide a form of visual representation of the MI task as well as to act as the NF signal.

## Materials and Methods

### Participants

Twenty two healthy young volunteers (11 males and 11 females) were recruited for this study. The participants’ age ranged from 20 to 33 years old (mean age = 23.18 years, standard deviation = 3.5 years). All participants had no history of neurological or psychiatric disorders, right handed as indexed by a handedness inventory test, with Mini-Mental State Examination scores greater than 28, and had no prior experience with real-time fMRI or the MI tasks. This study was approved by the Institutional Review Board of the National Center for Geriatrics and Gerontology of Japan (Protocol #938; Title: Development of cognitive function measurement system using real-time fMRI; Approval date: June 19, 2016). Written informed consent was obtained from all participants before joining the study.

### Experimental Paradigm and Tasks

All participants underwent two MI training sessions, one with NF and the other without NF (non-NF), 7 or 11 days apart. Due to this design, participants were divided into two groups. Group A participants (*N*_A_ = 11) started with the non-NF session followed by the NF session. On the other hand, group B participants (*N*_B_ = 11) started with the NF session followed by the non-NF session. Participants were randomly assigned to each group.

Each training session consisted of the following scans: (1) an anatomical localizer run, (2) 3D MPRAGE (Magnetization Prepared Rapid Acquisition Gradient Echo, Siemens) scan for a reference anatomical image, and (3) 4 task-based functional MRI scans. The task scans consisted of 9-rest and 8-task blocks alternated with each other with each block lasting for 30 s. The task blocks were divided into 4 blocks of imagined left hand gripping and opening (LGO) and another 4 blocks of imagined right hand gripping and opening (RGO). The task fMRI scans during NF sessions included a pre-feedback scan (run 0) with no NF and 3 NF scans (runs 1 – 3). For non-NF sessions, the participants performed the same task in all 4 scans (runs 0 – 3) with no NF. Participants were given a 5-min break in between scans.

### Imaging Parameters

Both functional and anatomical scans were acquired using a Siemen’s Magnetom Trio (Siemens, Erlanger, Germany) 3.0T scanner with a 12-channel head coil. T1-weighted MR images were acquired using a 3D MPRAGE pulse (Mugler and Brookeman, 1990) sequence for anatomical reference with the following imaging parameters: repetition time (TR) = 2.53 s, echo time (TE) = 2.64 ms, 208 sagittal slices with a 50% distance factor and 1mm thickness, field of view (FOV) = 250 mm, 256 × 256 matrix dimension, and in-plane voxel resolution of 1.0 mm × 1.0 mm. Functional MR images were acquired using a gradient echo (GE) echo planar imaging (EPI) sequence with the following imaging parameters: TR = 2.0 s, TE = 30 ms, flip angle (FA) = 80°, 37 axial slices with a distance factor of 30%, FOV = 192 mm, slice thickness = 3.0 mm, 64 × 64 matrix dimension, voxel size = 3.0 mm × 3.0 mm × 3.0 mm, and a total of 255 volumes.

### Real-Time Neurofeedback System

**FIGURE 1:**
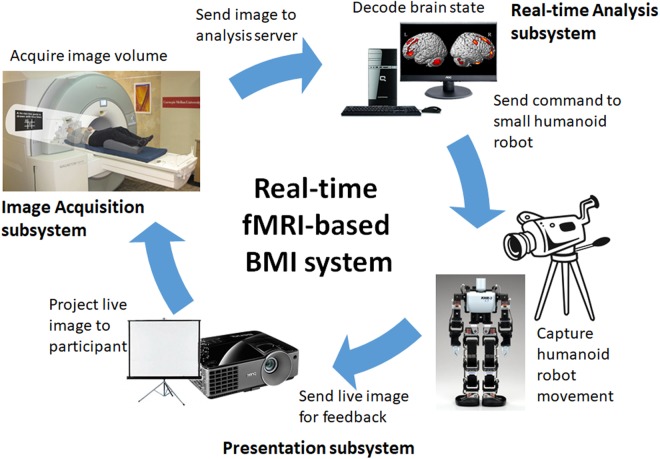
Schematic representation of the real-time neurofeedback system consisting of an image acquisition subsystem, a real-time analysis subsystem, and a presentation subsystem.

The schematic representation of our real-time neurofeedback system is shown in **Figure 1**. It consists of three subsystems, namely (1) image acquisition subsystem, (2) real-time analysis subsystem, and (3) presentation subsystem. The image acquisition subsystem consists of the MRI scanner and its console and is responsible for MR image acquisition, real-time image reconstruction, and real-time image transfer to the analysis subsystem. The real-time analysis subsystem, consisting of a dedicated workstation running the Linux operating system, is responsible for the real-time analysis of the acquired images including image preprocessing, statistical analysis, and brain state decoding/classification, among others. The presentation subsystem is responsible for input stimuli as well as feedback presentation. Currently, it supports screen-projector combination for simple stimuli and feedback presentation as well as video camera-small humanoid robot (KHR-3V, Kondo Science, Japan) combination for BMI applications.

At present, the real-time fMRI-based BMI system enables us to control the arm movements (e.g., raising or lowering) of the humanoid robot using MI tasks. The system operates as follows (**Figure 1**). During real-time operation, the image acquisition subsystem sends the acquired functional images volume-by-volume to the analysis subsystem, which then processes the data. After processing and if the target brain activation pattern has been identified, a command (e.g., raise left arm) is sent to the humanoid robot via a USB cable connection. The robot then executes the movement associated with the command signal. The action of the robot is captured by a video camera, which sends live video feed to the participant via a projector for feedback. This enables the participant to control the arm movements of the humanoid robot by consistently generating brain activation patterns associated with the target MI task.

### Support Vector Machines

To identify the brain activation patterns associated with the different MI tasks, we used support vector machines (SVMs) for both real-time and offline analyses. SVM is a supervised machine learning algorithm used for classification or regression analysis. It requires training examples to generate a classification model for a given problem. In general, given two classes of objects, SVM attempts to determine a separating hyperplane (decision boundary) optimizing the separation between the two groups using the provided training samples. The obtained model can then be used to classify new samples not yet seen by the SVM algorithm. In all SVM analyses, we used a linear SVM algorithm and set the regularization parameter *c* to 1. All analyses were performed in Matlab using in-house scripts and LIBSVM^1^ (Chang and Lin, 2011).

### Neurofeedback Training

During NF sessions, data from the pre-feedback scan (run 0) were used to train SVMs online to classify the different brain activation patterns associated with the MI tasks. Here, we used several SVM classification models including Rest vs. LGO (to classify image volumes during rest blocks and LGO task blocks), Rest vs. RGO, and LGO vs. RGO. The trained SVMs were then used in the succeeding NF scans to classify the acquired functional images in real time and to generate the corresponding BMI commands to control the arm movement of the small humanoid robot connected to the real-time fMRI system. The robot’s whole body was projected on a screen by the system’s video camera to provide visual feedback to the participants. Before the start of each NF scan, the robot’s arms were initially set in a horizontally extended position. For each image volume, correct task classification of the SVM would generate a BMI command that would initially raise either the left (for LGO tasks) or the right (for RGO tasks) arm of the robot by about 85° from the horizontal and then lower the arm back to its initial horizontal position before the next volume was acquired, thus providing a continuous feedback during task blocks. For incorrect classification, the robot would remain stationary. Note that during rest blocks and for non-NF scans (run 0 for NF session and runs 0 – 3 for non-NF session), the robot was stationary with horizontally extended arms and the participants focused their attention on a cross mark positioned on the robot’s body.

### Real-Time Image Preprocessing

Data from the pre-feedback scan during NF sessions were preprocessed immediately after the scan (online analysis) using SPM8 (Wellcome Trust Center for Neuroimaging, London, United Kingdom). Functional images were realigned relative to the scan’s mean functional image, *I*_mean_. Realigned images were then smoothed using an 8-mm full-width-at-half-maximum (FWHM) 3D Gaussian kernel. These preprocessed images were used to train the SVMs. A brain mask was also created to exclude voxels outside the brain in the classification analysis. To do this, we used SPM’s normalization procedure to find a transformation from the standard MNI (Montreal Neurological Institute) space to the subject space using an EPI template in MNI space and *I*_mean_. We then applied the obtained transformation to a brain mask in MNI space to transform it to the subject space. The normalized mask was further co-registered to *I*_mean_, then re-sliced to have the same dimension and orientation as *I*_mean_. During feedback scans, each functional image was realigned to *I*_mean_ immediately after acquisition, smoothed using an 8-mm FWHM Gaussian kernel, masked to exclude outside brain voxels, and used as input to the trained SVMs for the real-time classification of brain activation patterns. We also applied an incremental detrending approach to the real-time output of the classifier to correct for possible classifier drift (LaConte et al., 2007).

### Offline Data Analysis

Since there was no feedback and no real-time brain activation pattern classification during non-NF sessions, we analyzed data sets from both NF and non-NF sessions offline and comparisons were performed based on these analyses. For the offline analysis of all imaging data, we used SPM12. T1-weighted images were first segmented into component images including gray matter, white matter, cerebrospinal fluid, and non-brain tissues using SPM’s segmentation approach (Ashburner and Friston, 2005). For the functional data, the first 5 volumes were discarded to account for the initial image inhomogeneity. Functional images were then realigned relative the mean image from the series, co-registered to the bias-corrected anatomical image obtained from the segmentation step, normalized to standard MNI space, resampled to an isotropic voxel resolution of 2 mm × 2 mm × 2 mm, and spatially smoothed using an 8-mm FWHM 3D Gaussian filter.

To identify brain regions activated during each task, we used a box-car convolved with the canonical hemodynamic response function to model each MI task. The 6 estimated motion parameters were also included in the model as nuisance regressors to account for head motion. Contrast images were extracted for each task and group results were obtained using a one-sample *t*-test with the contrast images as inputs. To compare differences in activation between NF and non-NF sessions, we performed a paired sample *t*-test for each run using the contrast images from the first level analysis as inputs. We also used a 2 × 3 factorial analysis with within-subject factors of NF (with and without) and training runs (runs 1–3) using the flexible factorial design implemented in SPM12 to investigate the overall difference in activation patterns between NF and non-NF sessions (main effect of NF). Additionally, a one-way within-subject analysis of variance (ANOVA) was also performed to compare activation changes across NF runs during NF session. Activation maps were generated using a threshold value of *p* < 0.05 corrected for multiple comparisons using family-wise error cluster level correction (FWEc) with cluster defining threshold (CDT) set to *p* = 0.001. We used the Neuromorphometrics atlas available in SPM12 to label the different cortical areas in the obtained statistical maps.

We also performed offline SVM analyses using the preprocessed data sets. For each participant, data from run 0 were used to train the SVMs and the resulting classification models were then tested using data from runs 1 – 3. Separate SVMs were trained for scans from NF and non-NF sessions. To evaluate the SVMs’ performance, we computed the task predictive value (TPV) defined here as the ratio between the number of task volumes correctly classified and the total number of task volumes as well as accuracy defined as number of correctly classified volumes over the total number of volumes. We used TPV in evaluating the performance of classification models comparing rest and task volumes (i.e., Rest vs. LGO and Rest vs. RGO) since rest blocks were not monitored and participants might have practiced the task during rest blocks that could lead to inaccurate classification. On the other hand, for LGO vs. RGO classification model, we used the classification accuracy.

## Results

### Brain Activation During MI Tasks

**FIGURE 2:**
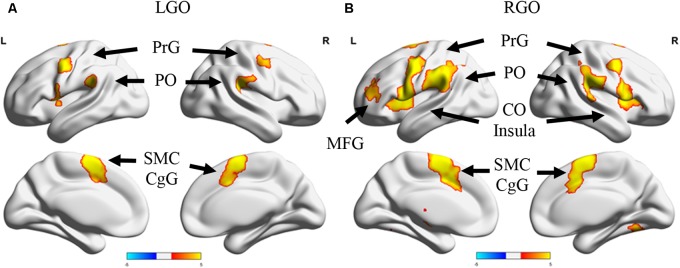
Activation maps for run 0 during the first session. Surface projections of the activation maps showing regions that were activated during **(A)** imagined left hand gripping and opening (LGO) and **(B)** imagined right hand gripping and opening (RGO). Activation maps were generated using a threshold value of *p* < 0.05 corrected for multiple comparisons using family wise error correction at the cluster level. Note that some regions in the cerebellum were also activated but were not shown in the figure. PrG, precentral gyrus; PO, posterior operculum; CO, central operculum; SMC, supplementary motor cortex; CgG, cingulate gyrus; MFG, middle frontal gyrus.

**Figure 2** shows the group activation maps using all participants associated with the two MI tasks in run 0 during the first session. Compared to rest, LGO showed activation in bilateral supplementary motor cortex (SMC) spreading toward the right cingulate gyrus (CgG), bilateral cerebellum (not shown), bilateral precentral gyrus (PrG), bilateral parietal operculum (PO), and left central operculum (CO). On the other hand, RGO showed activations in bilateral SMC extending toward CgG, bilateral PrG extending toward inferior frontal gyrus (IFG)/Brodmann area (BA) 44 and insula/BA13, bilateral putamen, bilateral inferior parietal lobule (IPL), bilateral cerebellum, right thalamus/caudate, and left middle frontal gyrus (MFG). The list of activated regions with the corresponding cluster peak MNI coordinates, cluster sizes, and *z*-values is shown in **Table 1**.

**Table 1:** List of activated regions for imagined left and right hand gripping and opening during the 1st scan session.

	Peak location MNI (mm)			Cluster size	*z*-value	Cortical area
			
	*X*	*Y*	*Z*			
LGO	—8	2	58	2256	5.75	L SMC
	38	-54	-28	277	4.79	R Cer
	-50	6	4	680	4.7	L CO
	52	4	46	439	4.56	R PrG
	-50	-2	46	531	4.52	L PrG
	-42	-38	24	463	4.45	L PO
	52	-32	24	678	4.42	R PO
	-30	-60	-24	420	4.32	L Cer
RGO	—6	2	62	3304	5.83	L SMC
	52	2	46	2533	5.72	R PrG
	-48	-6	44	7466	5.49	L PrG
	32	-62	-22	2578	5.34	R Cer
	54	-34	22	1659	5.14	R PT/PO
	22	-14	20	433	3.93	R Th
	-34	50	20	524	3.9	L MFG


*Activation maps were generated using a threshold value of *p* < 0.05 corrected for multiple comparisons using family-wise error correction at the cluster level. LGO, imagined left hand gripping and opening; RGO, imagined right hand gripping and opening; SMC, supplementary motor cortex; Cer, cerebellum; CO, central operculum; PrG, precentral gyrus; PO, parietal operculum; PT, planum temporal; Th, thalamus; MFG, middle frontal gyrus; L, left; R, right.*

**FIGURE 3:**
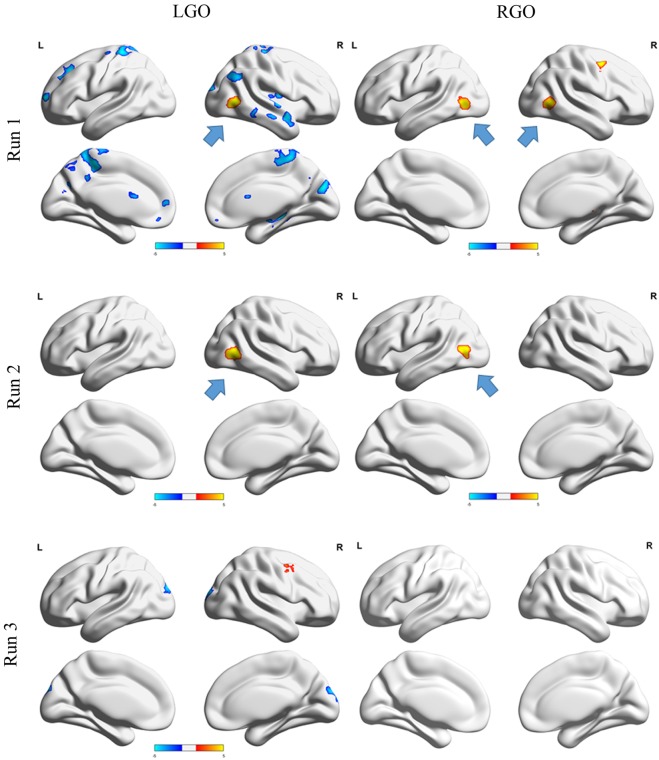
Contrast maps comparing NF and non-NF sessions for runs 1 **(top row)**, 2 **(middle row)** and 3 **(bottom row)** for LGO **(left column)** and RGO **(right column)** tasks. Regions where NF > non-NF are shown in red-yellow colors, whereas regions where NF < non-NF are shown in dark-light blue colors. Contrast maps were generated using a threshold value of *p* < 0.05 corrected for multiple comparisons using family wise error correction at the cluster level. Blue arrows indicate the location of left or right middle temporal gyrus/inferior occipital gyrus. LGO, imagined left hand gripping and opening and RGO, imagined right hand gripping and opening.

Contrast maps showing significant run-specific differences in activation during NF and non-NF sessions are shown in **Figure 3**. LGO task activated more regions during non-NF session than NF session in runs 1 and 3. These regions included the right cerebellum, bilateral postcentral gyrus (PoG), left superior frontal gyrus (SFG), right superior temporal gyrus (STG), right posterior insula (PIns), and right hippocampus in run 1 and the left superior occipital gyrus (SOG) in run 3. The only region that exhibited stronger activation during NF session was the right inferior occipital gyrus (IOG)/middle temporal gyrus (MTG) in run 1, which persisted even in run 2. For the RGO task, the right PrG and the left MTG/IOG were activated strongly during NF in run 1. The stronger activity in left MTG/IOG persisted even in run 2. Finally, in run 3, no significant differences were observed between the two sessions. The list of regions showing significant difference in activation between NF and non-NF sessions is given in **Table 2**.

**Table 2:** List of regions showing significant difference in activations between NF and non-NF sessions using paired sample *t*-tests.

	Peak location MNI (mm)			Cluster size	*z*-value	Cortical area
				
	*X*	*Y*	*Z*			
LGO – run 1						
NF > non-NF	46	-66	2	374	4.47	R IOG
NF < non-NF	34	-76	-30	561	5.00	R Cer
	-18	-46	64	2601	4.72	L PoG
	48	4	-26	315	4.45	R STG
	-22	58	10	365	4.42	L SFG
	-26	40	46	417	4.40	L SFG
	32	-18	14	417	4.40	R PIns
	28	-34	—6	460	4.23	R Hip
	6	-80	26	475	4.20	R Cun
	-20	4	28	367	4.13	L Cau
	54	-36	-16	236	3.96	R ITG
	54	-58	34	213	3.71	R AnG
RGO – run 1						
NF > non-NF	8	-22	8	257	4.89	R Th
	42	0	52	343	4.78	R PrG
	-42	-66	4	415	4.59	L MTG
	44	-62	0	287	4.19	R MTG
LGO – run 2						
NF > non-NF	48	-62	2	484	5.43	R MTG
RGO – run 2						
NF > non-NF	-40	-72	8	494	4.90	L IOG
LGO – run 3						
NF > non-NF	38	0	44	222	4.59	R PrG
NF < non-NF	18	-94	16	436	4.10	R OCP


*Contrast maps were generated using a threshold value of *p* < 0.05 corrected for multiple comparisons using family-wise error correction at the cluster level. IOG, inferior occipital gyrus; Cer, cerebellum; PoG, postcentral gyrus; STG, superior temporal gyrus; SFG, superior frontal gyrus; PIns, posterior insula; Hip, hippocampus; Cun, cuneus; Cau, caudate; ITG, inferior temporal gyrus; AnG, angular gyrus; Th, thalamus; PrG, precentral gyrus; MTG, middle temporal gyrus; OCP, occipital pole; L, left; R, right.*

**FIGURE 4:**
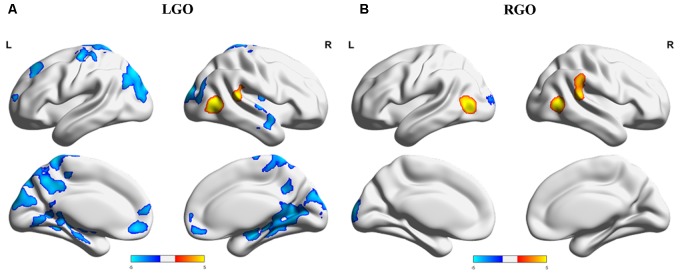
Overall difference in activation between NF and non-NF sessions (main effect of NF in the factorial analysis) for **(A)** LGO and **(B)** RGO tasks. Regions where NF > non-NF are shown in red-yellow colors, while regions where NF < non-NF are shown in dark-light blue colors. Statistical maps were thresholded using FWEc *p* < 0.05. LGO, imagined left hand gripping and opening and RGO, imagined right hand gripping and opening.

Overall differences in activation between NF and non-NF (main effect of NF in the factorial analysis) for the two MI tasks are shown in **Figure 4**. Consistent with run-specific results, LGO task activated more regions during non-NF session than NF session including a very large cluster with peak location in the right MTG and extending toward several other regions including left cuneus, right occipital pole (OCP), left PrG, and left PoG, among others, right cerebellum, left medial frontal cortex (MFC), and left SFG. Regions showing significant activation during NF included right MTG and right STG. For the RGO task, the left IOG, right MTG, and right STG showed significant activation during NF, while the left OCP showed significant activation during non-NF. Comparisons across runs during NF (one-way within-subject ANOVA) did not show any significant differences in activation. The full list of regions showing significant difference in activation between NF and non-NF sessions is given in **Table 3**.

**Table 3:** List of regions showing overall (main effect of NF) differences in activation between NF and non-NF sessions.

	Peak location MNI (mm)			Cluster Size	*z*-value	Cortical area
				
	*X*	*Y*	*Z*			
RGO						
NF > non-NF	-42	-70	4	725	6.54	L IOG
	50	-64	4	533	5.6	R MTG
	64	-36	18	667	5.28	R STG
NF < non-NF	-8	-98	18	363	4.85	L OCP
LGO						
NF > non-NF	48	-64	6	769	7.53	R MTG
	62	-38	16	380	5.13	R STG
NF < non-NF	44	-6	-24	12661	5.41	R MTG
	-6	46	-10	842	4.5	L MFC
	-24	40	42	376	4.21	L SFG


*An FWEc *p* < 0.05 value was used to threshold all statistical maps. LGO, imagined left hand gripping and opening; RGO, imagined right hand gripping and opening; IOG, inferior occipital gyrus; MTG, middle temporal gyrus; STG, superior temporal gyrus; OCP, occipital pole; MFC, medial frontal cortex; SFG, superior frontal gyrus; L, left; R, right.*

### SVM Classification Performance

**FIGURE 5:**
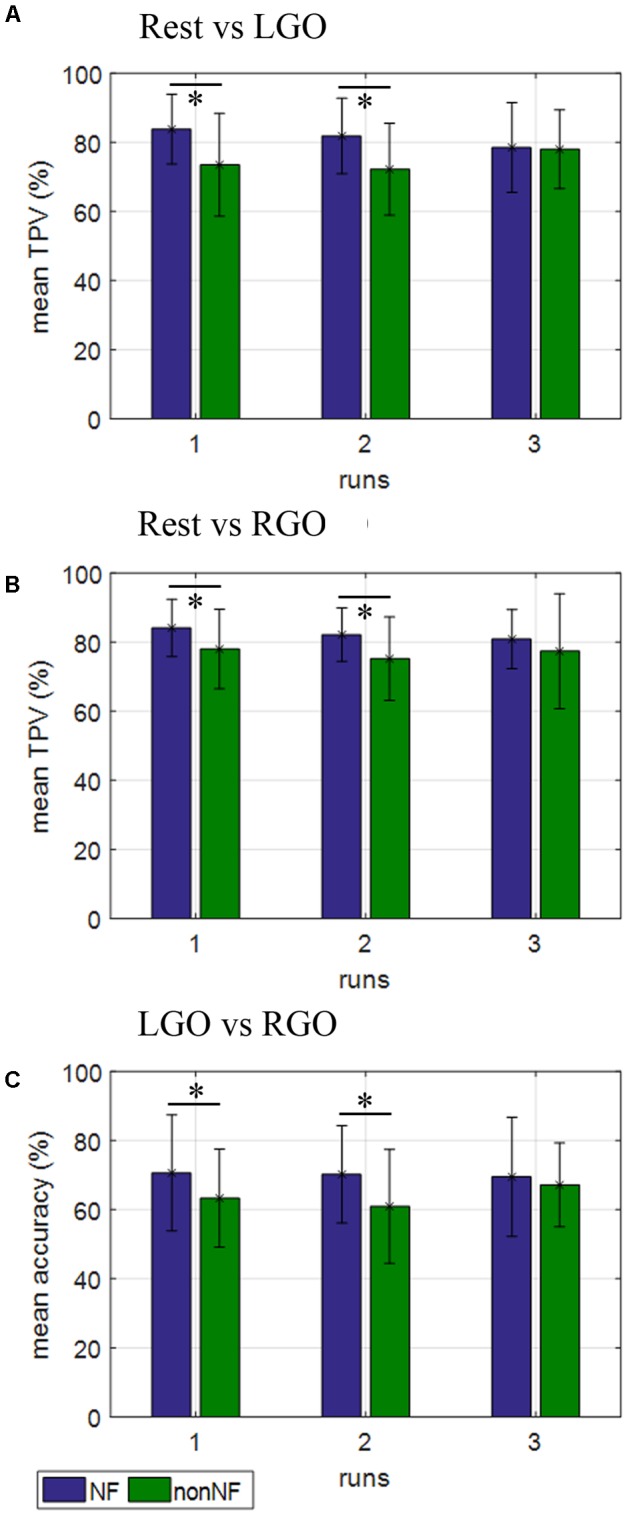
Support vector machines (SVMs) classification performance for **(A)** Rest vs. LGO, **(B)** Rest vs. RGO, and **(C)** LGO vs. RGO classification models during NF (blue bars) and non-NF (green bars) sessions. ^∗^Indicates significant difference in classification performance between NF and non-NF sessions.

The classification performance of the trained SVMs is shown in **Figure 5** and **Table 4**. For the classification models Rest vs. LGO (**Figure 5A**) and Rest vs. RGO (**Figure 5B**), the mean TPVs are plotted. On the other hand, for LGO vs. RGO (**Figure 5C**), the classification accuracies are shown. During NF scans, the overall average TPV for the 3 runs is about 81.36% for Rest vs. LGO and 82.35% for Rest vs. RGO and the overall average accuracy is 70.09% for LGO vs. RGO. During non-NF scans, the overall average TPV value is 74.54% for Rest vs. LGO and 76.82% for Rest vs. RGO and the average accuracy is 63.78% for LGO vs. RGO. No significant difference in TPVs and accuracies were observed across runs for both NF and non-NF sessions.

**Table 4:** Support vector machines classification performance.

	Rest vs. LGO (mean TPV)			Rest vs. RGO (mean TPV)			LGO vs. RGO (mean accuracy)		
			
	NF	Non-NF	*p*-value	NF	Non-NF	*p*-value	NF	Non-NF	*p*-value
ALL									
Run 1	83.79	73.48	0.0001	84.09	77.95	0.0134	70.61	63.30	0.0217
Run 2	81.82	72.20	0.0020	82.12	75.15	0.0106	70.19	60.91	0.0019
Run 3	78.48	77.95	0.8495	80.83	77.35	0.3161	69.47	67.12	0.3730
Group A									
Run 1	83.18	71.51	0.0057	86.21	75.30	0.0034	76.59	64.55	0.0093
Run 2	80.76	70.61	0.0250	83.33	71.67	0.0021	76.14	63.49	0.0001
Run 3	75.61	77.42	0.6603	79.85	78.64	0.7066	76.14	70.23	0.1252
Group B									
Run 1	84.39	75.45	0.0152	81.97	80.61	0.6578	64.62	62.05	0.5566
Run 2	82.88	73.79	0.0485	80.91	78.64	0.5491	64.24	58.33	0.2355
Run 3	81.36	78.48	0.4743	81.82	76.06	0.3695	62.80	64.02	0.7434


*Group A participants started with non-NF session followed by NF session while group B participants did the opposite, first with NF session followed by the non-NF session. Shown *p*-values are from paired sample *t*-test comparing the performance between NF and non-NF sessions.*

Comparing NF and non-NF using paired sample *t*-tests, TPV during NF was significantly higher than that during non-NF in run 1 (*p* = 0.0001) and run 2 (*p* = 0.002) but not in run 3 (*p* = 0.8495) for Rest vs. LGO and also in run 1 (*p* = 0.0134) and run 2 (*p* = 0.0106), but not in run 3 (*p* = 0.3161) for Rest vs. RGO. The same was true in terms of the classification accuracies for LGO vs. RGO, which was significantly higher in run 1 (*p* = 0.0217) and run 2 (*p* = 0.0019), but not in run 3 (*p* = 0.3730). This suggests that SVMs were able to classify brain activation patterns more reliably (higher classification performance) during NF sessions than during non-NF sessions.

**FIGURE 6:**
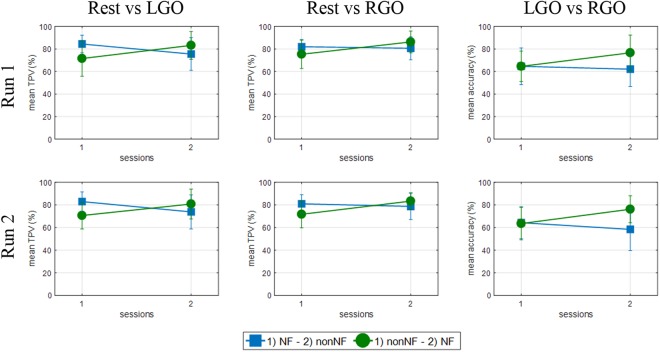
Support vector machines classification performance for the two groups of participants in runs 1 **(top row)** and 2 **(bottom row)** for the Rest vs. LGO **(left column)**, Rest vs. RGO **(middle column)** and LGO vs. RGO **(right column)** classification models. Group A (green circles) started with the non-NF session followed by the NF session, while Group B (blue squares) started with the NF session followed by the non-NF session.

We further investigated if previous experience in MI tasks could have affected the improvement in the classification performance during NF sessions since by design half of the participants (group A) did non-NF session, which provided the participants prior training of the MI tasks, before the NF session. Group A participants could drive the observed significant difference. The classification performance for the two MI tasks in runs 1 and 2 for each subgroup is shown in **Figure 6**. Group A (green circle) showed significant increase in TPV from session 1 to session 2 for both MI tasks (*p* = 0.0057 and *p* = 0.0250 in runs 1 and 2, respectively, for LGO and *p* = 0.0034 and *p* = 0.0021 in runs 1 and 2, respectively, for RGO). Classification of the two tasks (*p* = 0.0093 and *p* = 0.0001 in runs 1 and 2, respectively, for LGO vs. RGO) also became more accurate in the second session with NF. On the other hand, for group B (blue squares), we observed a significant decrease in TPV (*p* = 0.0152 and *p* = 0.0485 in runs 1 and 2, respectively) for the LGO task when no feedback is given (session 2), while no significant change was observed in both TPV for RGO and classification accuracy for LGO vs. RGO. Moreover, group B also had a relatively higher TPV in the first session compared to group A. In run 3, we did not observe any significant difference in the classification performance of the trained SVM between sessions and groups and so was not included in the figure. These results further reinforced the important role of NF in generating consistent brain activation patterns during MI task training.

## Discussion

In this study, we investigated the importance of NF in generating consistent brain activation patterns during an extended MI task training using a BMI system to provide a form of visual feedback of the MI tasks. Our results confirmed our hypothesis that participants were able to consistently reproduce brain activation patterns associated with the MI task when provided with NF than without NF as measured by the classification performance of SVMs trained to identify MI-related activation patterns. Moreover, these findings also demonstrated the capability of participants to volitionally recall MI-related brain activation patterns in the presence of contingent feedback. The improvement in the classification performance during NF was also associated with the activation of MTG/IOG suggesting the significance of performance monitoring in generating consistent brain activation.

Previous neuroimaging studies have demonstrated the importance of NF, based on real-time fMRI, in the training of participants to self-regulate brain activity in circumscribed brain regions (Posse et al., 2003; deCharms et al., 2005; Caria et al., 2007, 2010; Sulzer et al., 2013). More recent studies have extended the application of NF to voluntary control of the connectivity between brain regions (Koush et al., 2013, 2015) or induction of multi-voxel activation patterns (Shibata et al., 2011). Our findings have demonstrated the use of NF for the improvement of volitional recall of specific activation patterns (in this case, MI-related brain activation patterns). This is important, for example, in BMI applications where successful recall of activation patterns could provide consistent and improved BMI operation. For neurorehabilitation, volitional recall may also prove useful in re-acquiring learned task-related activations disrupted by the insult to the brain. It has also the potential to promote long-term learning by stabilizing activation patterns via repeated retrieval or recall of relevant activation patterns. To establish the relevance and efficacy of this NF application, more detailed application-specific studies will be necessary.

Our finding showing the efficacy of MI training with NF compared to non-NF is consistent with the result of Zich et al. (2015) in a study using EEG-based feedback during simultaneous EEG-fMRI. Using a block design (2 blocks without NF and 1 block with NF), the authors observed stronger MI-related activation in contralateral regions in both EEG and fMRI BOLD signals when NF was provided. Most recently, Perronnet et al. (2017) compared unimodal EEG-based NF and fMRI-based NF with bimodal EEG-fMRI-based NF. Their results again showed significantly higher activation during MI with NF than without NF. Interestingly, NF involving fMRI appeared to have higher activation compared with unimodal EEG-based NF. But unlike these studies where NF and non-NF tasks were done in the same scan or same session, we divided NF and non-NF tasks into two separate sessions and used extended practice runs (3 runs) for both tasks to simulate actual training. Thus, our findings further demonstrated the benefits of NF even for an extended MI task training.

In addition, our result also showed that without NF prior experience of MI does not necessarily translate to improvement in performance (**Figure 6**). Comparing the two groups of participants, performance of those that started without NF (Group A) significantly increased in the second session with NF. At first, this finding may suggest the effect of prior MI experience for improved performance. However, participants in Group B did not show any performance improvement in the second session when no NF was provided. In fact, the initial performance in the session with NF was even better than in the second session without NF. This is particular more evident in the LGO vs. RGO classification result.

Compared to many BCI/BMI approaches, we used real-time fMRI instead of EEG. The main advantage of real-time fMRI-based NF is that the NF signal is derived directly from the activation pattern of the relevant cortical regions. Changes in the NF signal are therefore directly related to the activity changes in these regions (e.g., Posse et al., 2003; deCharms et al., 2005; Caria et al., 2007; Hamilton et al., 2016). Other works had also successfully used MI to control activation in specific motor and somatosensory regions (Yoo and Jolesz, 2002; DeCharms et al., 2004; Yoo et al., 2008) using NF signal derived from these regions. By contrast, EEG-based NF commonly used the spontaneous modulation of global brain oscillation such as the desynchronization of the sensorimotor rhythm. Due to the inverse problem inherent in EEG source reconstruction, changes in the measured brain oscillatory pattern cannot be uniquely attributed to changes in activity patterns in specific areas within the sensorimotor network. However, EEG has the advantage in terms of costs and portability (Kranczioch et al., 2014) and is therefore more accessible for practical use. Thus, a combined use of EEG-based NF and real-time fMRI-based NF may prove optimal for a successful MI training approach.

Unlike previous real-time fMRI-based NF studies involving MI (Yoo and Jolesz, 2002; DeCharms et al., 2004; Yoo et al., 2008), we used a multivariate pattern classification approach (LaConte et al., 2007) in the training sessions to generate NF signals during MI training. This approach ensures that the feedback is based on the activation pattern of distributed MI-related cortical regions (Grèzes and Decety, 2000; Simos et al., 2017) rather than on the activity of a single region-of-interest (ROI). This is particularly relevant when the tasks being considered, such as MI, involved overlapping activation regions. Using activation patterns instead of the strength of ROI activation as basis for NF could improve the specificity of the individual task. As an example, both LGO and RGO tasks activated SMC and bilateral PrG and therefore could not be effectively differentiated by the activation of these regions. However, the overall activation patterns differed between the two tasks, and therefore could be more useful in improving the classification of the two tasks. By using patterns of activations over multiple brain regions, the BMI system could provide more relevant NF signal to the participants than just by using the strength of activation of a single ROI.

In terms of brain activation, we observed greater activation in IOG/MTG during NF sessions. Activation in this region was also observed in previous studies involving EEG-based BCI NF (Marchesotti et al., 2017), real-time fMRI-based NF (Berman et al., 2012), and motor observation (Grèzes and Decety, 2000; Simos et al., 2017). Moreover, IOG/MTG activation was also associated with the perception of visual motion and visually guided motion (Astafiev et al., 2004; Arzy et al., 2006; Ninaus et al., 2013). As most NF involves some form of visual motion, the activation of this region could be associated with task performance monitoring, which in turn could provide guidance to help participants generate the correct brain activation patterns associated with the task during training.

We also note that a change in the classification performance (no significant difference between NF and non-NF session) and in the activation of the IOG/MTG was observed in run 3 of the NF session. There are two possibilities that may explain this change. The first possibility is fatigue. Participants had been performing the same tasks 4 times in the same scanning session with limited rest period in between scans. When participants were interviewed after the session, several did complain of being tired doing the MI tasks. To overcome this problem, training can be performed on multiple sessions separated by several days (deCharms et al., 2005). The other possibility is the effect of learning itself. Using a motor learning task, LaConte (2011) showed that prediction accuracy decreased for participants who demonstrated motor learning, whereas the opposite was true for those exhibiting little or no motor improvement. Learning is non-stationary and can introduce changes in the observed activation patterns. In the current context, after continuous practice with NF, participants were becoming more familiar with the task possibly resulting in changes in brain activation pattern, which in turn, led to a decrease in the classification performance of the trained SVMs. One way to mitigate this issue is to re-train the SVMs using the most recently acquired data to adapt the classifier to changes in the participants’ brain activation pattern. However, caution should be exercised when using this approach since if participants are not learning correctly, adapting SVMs to the most recent data sets will have negative effects. Another alternative is to pre-train an SVM using data sets that truly capture the target brain activation pattern and to use the trained classifier for all participants. The data set could come from an expert in MI or from a trained group of participants. With this, better classification performance can be easily interpreted to mean activation patterns closer to the desired target pattern.

Finally, the number of participants (*N* = 22) recruited for the study may also present some limitations. Although for the current purpose this number is already sufficient, investigation for differences in strategies used during MI task training, for example, may require more participants. Moreover, this also constraints us to employ the same group of participants for both NF and non-NF sessions and as a consequence, we had to take into account the potential effect of prior MI experience in the reported TPVs or accuracies. Although we have demonstrated that prior experience of MI did not affect the reported results, the smaller number of participants per group (*N* = 11) did limit us to further examine potential differences in brain activation patterns between the two groups.

## Conclusion

In summary, we have demonstrated the advantages of using NF during MI task training using a BMI system that provided a form of visual representation of the MI task and also acted as visual feedback. Our results showed that SVM’s classification performance was consistent across the three feedback runs. Higher TPVs/accuracies during NF sessions compared to non-NF sessions suggest that participants were able to maintain consistent activation patterns associated with the MI tasks in the former than in the latter. This improvement in the classification performance with NF was also associated with the additional activation in the left/right MTG/IOG during NF sessions, which could be related to visual motion processing as a means of tracking performance during NF scans. These findings corroborate previous studies showing the importance of NF in improving MI performance and also demonstrate the potential use of real-time fMRI-based NF to improve volitional recall of relevant brain activation patterns during MI task training.

## Author Contributions

EB, AY, MU, SK, HI, and TN conceived and designed the study. SK and KT built and programmed the humanoid robot for real-time fMRI application and EB developed the real-time analysis system. EB, AY, MU, and KT performed the experiments and analyzed the data. TN gave technical support and supervised the whole research procedure. EB, HI, and TN wrote the draft of the manuscript and all authors reviewed and approved the final version of the manuscript.

## Conflict of Interest Statement

The authors declare that the research was conducted in the absence of any commercial or financial relationships that could be construed as a potential conflict of interest.

## Footnotes

1 http://www.csie.ntu.edu.tw/~cjlin/libsvm
